# Examining the relationship between maternal body size, gestational glucose tolerance status, mode of delivery and ethnicity on human milk microbiota at three months post-partum

**DOI:** 10.1186/s12866-020-01901-9

**Published:** 2020-07-20

**Authors:** Lauren LeMay-Nedjelski, James Butcher, Sylvia H. Ley, Michelle R. Asbury, Anthony J. Hanley, Alex Kiss, Sharon Unger, Julia K. Copeland, Pauline W. Wang, Bernard Zinman, Alain Stintzi, Deborah L. O’Connor

**Affiliations:** 1 Department of Nutritional Sciences, University of Toronto, Medical Sciences Building, 1 King College Circle, Toronto, ON M5S 1A8 Canada; 2 Peter Gilgan Centre for Research and Learning, Translational Medicine, The Hospital for Sick Children, 686 Bay Street, Toronto, ON M5G 0A4 Canada; 3 Department of Biochemistry, Microbiology and Immunology, Ottawa Institute of Systems Biology, University of Ottawa, 451 Smyth Road, Ottawa, ON K1H 8M5 Canada; 4 Department of Epidemiology, Tulane University School of Public Health and Tropical Medicine, 1440 Canal Street, Suite 2001, Mail Box 8318, New Orleans, LA 70112 USA; 5 Department of Research Design and Biostatistics, Sunnybrook Research Institute, 2075 Bayview Ave, Toronto, ON M4N 3M5 Canada; 6 Department of Pediatrics, Mount Sinai Hospital, 600 University Ave, Toronto, ON M5G 1X5 Canada; 7 Department of Pediatrics, University of Toronto, Medical Sciences Building, 1 King College Cir, Toronto, ON M5S 1A8 Canada; 8 Lunenfeld-Tanenbaum Research Institute, Mount Sinai Hospital, University of Toronto, 600 University Ave, Toronto, ON M5G 1X5 Canada; 9 Centre for the Analysis of Genome Evolution and Function, University of Toronto, 25 Willcocks Street, Toronto, ON M5S 3B2 Canada

**Keywords:** Human milk, Microbiota, Body mass index, Gestational diabetes, Impaired glucose tolerance, Mode of delivery, Vaginal delivery, Caesarean delivery, Ethnicity, Microbiome

## Abstract

**Background:**

Few studies have examined how maternal body mass index (BMI), mode of delivery and ethnicity affect the microbial composition of human milk and none have examined associations with maternal metabolic status. Given the high prevalence of maternal adiposity and impaired glucose metabolism, we systematically investigated the associations between these maternal factors in women ≥20 years and milk microbial composition and predicted functionality by V4-16S ribosomal RNA gene sequencing (NCT01405547; https://clinicaltrials.gov/ct2/show/NCT01405547). Demographic data, weight, height, and a 3-h oral glucose tolerance test were gathered at 30 (95% CI: 25–33) weeks gestation, and milk samples were collected at 3 months post-partum (*n* = 113).

**Results:**

Multivariable linear regression analyses demonstrated no significant associations between maternal characteristics (maternal BMI [pre-pregnancy, 3 months post-partum], glucose tolerance, mode of delivery and ethnicity) and milk microbiota alpha-diversity; however, pre-pregnancy BMI was associated with human milk microbiota beta-diversity (Bray-Curtis R^2^ = 0.037). Women with a pre-pregnancy BMI > 30 kg/m^2^ (obese) had a greater incidence of Bacteroidetes (incidence rate ratio [IRR]: 3.70 [95% CI: 1.61–8.48]) and a reduced incidence of Proteobacteria (0.62 [0.43–0.90]) in their milk, compared to women with an overweight BMI (25.0–29.9 kg/m^2^) as assessed by multivariable Poisson regression. An increased incidence of *Gemella* was observed among mothers with gestational diabetes who had an overweight BMI versus healthy range BMI (5.96 [1.85–19.21]). An increased incidence of *Gemella* was also observed among mothers with impaired glucose tolerance with an obese BMI versus mothers with a healthy range BMI (4.04 [1.63–10.01]). An increased incidence of *Brevundimonas* (16.70 [5.99–46.57]) was found in the milk of women who underwent an unscheduled C-section versus vaginal delivery. Lastly, functional gene inference demonstrated that pre-pregnancy obesity was associated with an increased abundance of genes encoding for the biosynthesis of secondary metabolites pathway in milk (coefficient = 0.0024, *P*_FDR_ < 0.1).

**Conclusions:**

Human milk has a diverse microbiota of which its diversity and differential abundance appear associated with maternal BMI, glucose tolerance status, mode of delivery, and ethnicity. Further research is warranted to determine whether this variability in the milk microbiota impacts colonization of the infant gut.

## Background

Breastfeeding is the recommended method of feeding for all infants irrespective of whether the country of origin is low, middle, or high-income [1]. Human milk is a rich source of nutrients and bioactive components, such as the antimicrobial proteins lactoferrin and lysozyme, and contains an array of oligosaccharides which serve as a source of prebiotics [2–5]. It is now well accepted that human milk contains a rich supply of bacteria (~ 10^6^ bacterial cells/mL), which are believed to play an important role in postnatal colonization of the infant’s gastrointestinal tract (gut) [6–13]. Microbial composition of the gut, in turn, is associated with maturation of an infant’s gut and immune system, and aberrant gut microbial compositions have been linked to a number of short- and long-term health outcomes including diarrhea, respiratory tract infection, asthma, inflammatory bowel disease, obesity, and metabolic syndrome [14–18].

There is a limited understanding of the stability of the human milk microbiota in the face of environmental influences. Despite the high prevalence and known impact on other milk constituents, no study we are aware of has examined the association between maternal glucose tolerance status, either gestational diabetes or impaired glucose tolerance, and the milk microbiota. Only a few studies to date have cross-sectionally examined other maternal factors, such as maternal BMI and mode of delivery, on the microbiota composition of mature human milk (collected from 1-week to 6-months post-partum) [19–23]. Moreover, the findings from the few available studies are inconsistent likely due, in part, to the small number of study participants, and differing methods of milk collection and analysis (Additional file 1, Table S1). Of the limited studies conducted, maternal BMI and mode of delivery have been associated with the mature milk microbiota; however, many of these reports relied on small cohorts and thus their findings require replication in larger studies [19–23]. Further, most studies were unable to employ multivariable statistical modelling to adjust for multiple potential maternal factors of interest simultaneously. Lastly, none of these studies carried out functional inference analyses to determine if maternal BMI and mode of delivery also perturb the milk microbiome’s potential metabolic activities or predicted functional capabilities.

Therefore, we set out to fill these knowledge gaps in the field by taking advantage of milk samples and clinical metadata that had been previously collected from women enrolled in a prospective cohort study to investigate the impact of metabolic abnormalities and maternal nutrition in pregnancy on human milk composition (NCT01405547). The objective of this current study was to investigate the associations between maternal pre-pregnancy BMI (healthy, overweight, or obese), 3-month post-partum BMI, maternal glucose tolerance status in late pregnancy (gestational diabetes mellitus [GDM], impaired glucose tolerance [IGT], or normoglycemia), mode of delivery (vaginal delivery, unscheduled Caesarean delivery [C-section], or scheduled C-section), and ethnicity (white, Asian, or other [South Asian, Black, other]) on the microbial community composition and predicted functional capabilities of human milk at 3 months post-partum. This research will help to elucidate the modulatory potential of human milk microbiota in the face of physiological perturbations.

## Results

### Participant description

Milk samples were collected at 3 ± 1-month post-partum (mean ± standard deviation [SD]) (*n* = 113). Fifty-six (49.6%) mothers fed their infants their own milk exclusively at the time of milk collection (versus 57 [50.4%] mixed feeds), and 61 (53.9%) samples were from a complete breast expression (versus 52 [46.0%] from incomplete breast expression). The mean (± SD) age of the mothers was 34.2 ± 4.2 years (Table 1) with a pre-pregnancy BMI (kg/m^2^) of 24.3 ± 4.6, which is within a healthy BMI range (18.5–24.9 kg/m^2^). A modified oral glucose tolerance test (OGTT) administered at 30 weeks’ gestation (95% CI: 25–33 weeks) revealed that 24 (21.2%) women had GDM, 20 (17.7%) had IGT, and 69 (61.1%) had healthy glucose metabolism (normoglycemic). Among women with a healthy pre-pregnancy BMI, 12 and 16 had IGT and GDM, respectively. Among women with an overweight pre-pregnancy BMI, 5 had IGT and 5 had GDM (10 total). Lastly, among women with an obese pre-pregnancy BMI 3 had IGT and 3 had GDM (6 total; Additional file 2, Table S2). Sixty-four (56.6%) mothers delivered their infants vaginally, compared to the 49 (43.4%) who underwent a C-section.

**Table 1:** Baseline characteristics of mothers

Baseline variables	*n* = 113
Mean age (y), mean ± SD	34.2 ± 4.2
Ethnicity, No. (%)	
White	64 (56.6%)
Asian (Chinese, Korean, Japanese, Filipino)	27 (23.9%)
Other (South Asian, Black, other)	22 (19.5%)
Pre-pregnancy BMI^1^ (kg/m^2^),	
Mean ± SD	24.3 ± 4.6
Obese (> 30 kg/m^2^), No. (%)	11 (9.7%)
Overweight (25–29.9 kg/m^2^), No (%)	30 (26.5%)
Healthy (18.5–24.9 kg/m^2^), No (%)	72 (63.7%)
3-month post-partum BMI^1^ (kg/m^2^),	
Mean ± SD	26.4 ± 5.2
Obese (> 30 kg/m^2^), No. (%)	17 (15.0%)
Overweight (25–29.9 kg/m^2^), No. (%)	46 (40.7%)
Healthy (18.5–24.9 kg/m^2^), No. (%)	50 (44.2%)
Glucose tolerance status, No. (%)	
Gestational diabetes mellitus	24 (21.2%)
Impaired glucose tolerance	20 (17.7%)
Normoglycemic	69 (61.1%)
Mode of delivery, No. (%)	
Vaginal	64 (56.6%)
Scheduled Caesarean section	21 (18.6%)
Unscheduled Caesarean section	28 (24.8%)

^1^*BMI* Body mass index

### Overall microbial composition of human milk

**Fig. 1:**
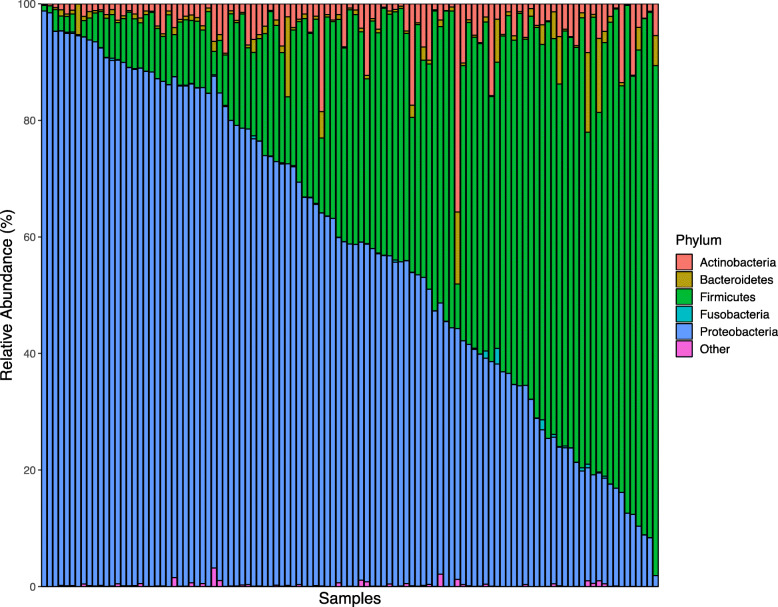
Microbial relative abundance in human milk at the phylum level (*n* = 109). The relative abundances of bacterial phyla in collected human milk samples are visualized using bar plots. For simplicity, only the most abundant 5 phyla are displayed with other phyla merged into the Other category

**Fig. 2:**
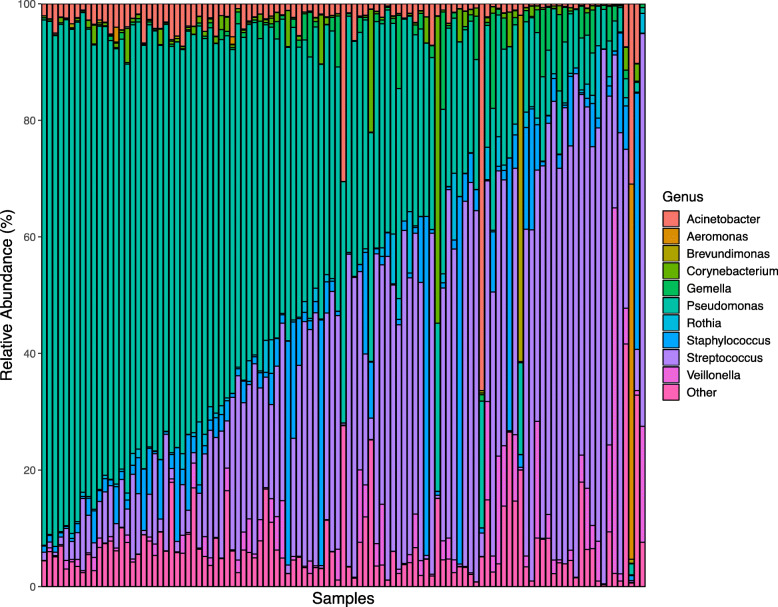
Microbial relative abundance in human milk at the genus level (*n* = 109). The relative abundances of bacterial genera in collected human milk samples are visualized using bar plots. For simplicity, only the most abundant 10 genera are displayed with other genera merged into the Other category

Following V4-16S rRNA gene sequencing, the average sequencing depth was found to be 63,025 (SD, 41,068) reads per sample. Data were rarefied to 20,000 reads per sample prior to calculating diversity metrics, and 4 samples were removed from analyses due to low sequence counts following filtering and rarefying (*n* = 109). Twenty-six unique phyla-level and 292 unique genus-level taxa were identified (Figs. 1 and 2). Proteobacteria and Firmicutes were the most abundant phyla at 58.6 ± 27.3% and 35.6 ± 26.3%, respectively, followed by Actinobacteria (4.1 ± 4.7%), Bacteroidetes (1.4 ± 2.7%), and Fusobacteria (0.1 ± 0.3%). *Pseudomonas* (43.4 ± 26.0%) and *Streptococcus* (30.6 ± 25.3%) were the predominant genera across all samples, followed by smaller abundances of *Staphylococcus* (6.2 ± 11.5%), *Acinetobacter* (3.5 ± 7.4%), *Veillonella* (3.2 ± 7.2%), *Gemella* (1.9 ± 3.3%), *Corynebacterium* (1.6 ± 5.5%), *Rothia* (1.3 ± 2.4%), *Aeromonas* (0.6 ± 6.2%), and *Brevundimonas* (0.6 ± 5.7%). Plotting the relative abundances of taxa at the phylum (Fig. 1) and genus (Fig. 2) taxonomic levels revealed obvious inter-individual variability in the microbial composition of milk. Of the top 5 phyla and top 10 genera, all taxa, with the exception of Fusobacteria and *Aeromonas,* were found in *>* 96% of all milk samples (Additional file 3, Table S3).

### Associations between maternal BMI, glucose tolerance status, mode of delivery, ethnicity and the milk microbiota

**Fig. 3:**
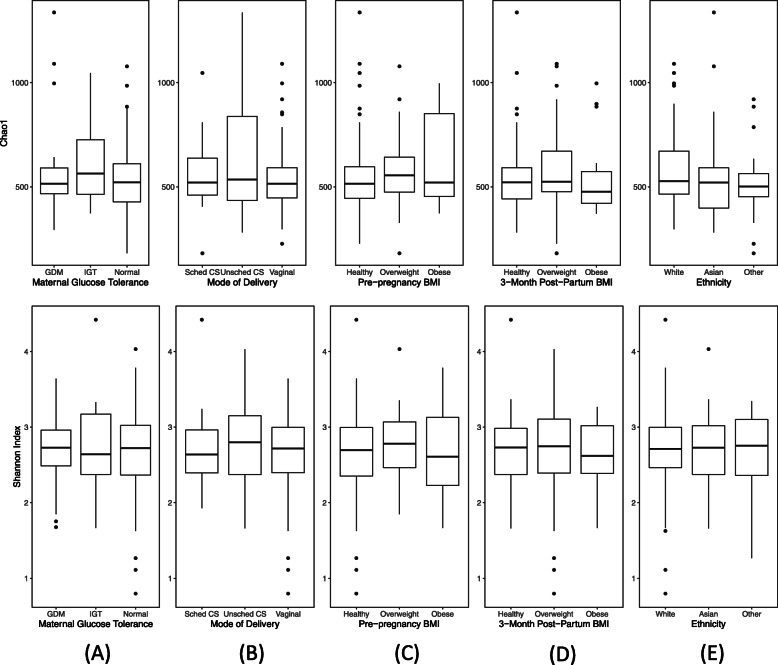
**a-e** The association between maternal characteristics and human milk microbiota alpha-diversity. The bacterial richness (Chao1 index) and diversity (Shannon index) of each human milk sample are plotted using box and whisker plots (mid-line = median; upper and lower bounds of the box = first and third quartile) as a function of **a** maternal glucose tolerance, **b** mode of delivery, **c** pre-pregnancy BMI, **d** 3-month post-partum BMI, **e** ethnicity. Multivariable linear regression analyses revealed no significant associations between the alpha-diversity of the milk microbiota and maternal metabolic and obstetrical characteristics. Abbreviations: GDM, gestational diabetes mellitus, IGT, impaired glucose tolerance; Sched CS, scheduled C-section; Unsched CS, unscheduled C-section

The Chao1 and Shannon indices were used to assess alpha-diversity (richness and diversity, respectively) within each human milk sample. No statistically significant associations were found between maternal characteristics (maternal glucose tolerance status, mode of delivery, pre-pregnancy BMI, 3-month post-partum BMI, ethnicity), and milk microbiota richness or diversity using multivariable linear regression analyses (Fig. 3a-e; results in Additional file 4, Table S4).

To further investigate associations between maternal characteristics and microbial composition, beta-diversity of the milk microbiota was assessed by principal coordinate analysis (PCoA) using the weighted UniFrac distance metric and the Bray-Curtis index of dissimilarity (Additional file 5, Fig. S1A-E; Additional file 6, Fig. S2A-E; Additional file 7, Table S5) [24]. No obvious clustering or separation based on maternal characteristics was observed; however, a small but statistically significant association between maternal pre-pregnancy BMI and beta-diversity clustering was identified (Bray-Curtis R^2^ = 0.037, *p* = 0.031). No other statistically significant associations were found for the other maternal characteristics and beta-diversity.

We assessed whether taxa abundance was associated with maternal characteristics by using multivariable Poisson regression models and accounting for multiple comparisons (Table 2; Additional file 8, Table S6; Additional file 9, Table S7; Additional file 10, Table S8; Additional file 11, Table S9). At least one maternal characteristic was associated with the differential abundance of Proteobacteria, Bacteroidetes, Firmicutes and Actinobacteria at the phylum level, and *Staphylococcus*, *Streptococcus*, *Pseudomonas*, *Veillonella*, *Gemella*, *Aeromonas*, *Corynebacterium* and *Brevundimonas* at the genus level.

**Table 2:** Associations between maternal characteristics and top 5 phyla and top 10 genera: Grouped by BMI

Taxa	Group effect ***p***-value	Pairwise comparison	IRR	95% CI	Pairwise comparison ***p***-value
**Pre-pregnancy BMI**					
**Phylum**					
Proteobacteria	0.019	Obese vs overweight	0.62	0.43–0.90	0.012
		Overweight vs healthy	1.23	1.00–1.50	0.045
Bacteroidetes	0.0051	Obese vs overweight	3.70	1.61–8.48	0.002
		Obese vs healthy	2.56	1.27–5.17	0.0086
**Genus**					
*Staphylococcus*	0.011	Obese vs overweight	2.50	1.09–5.72	0.031
		Obese vs healthy	3.15	1.47–6.76	0.0032
*Corynebacterium*	0.0003	Obese vs overweight	5.13	1.79–14.70	0.0023
		Obese vs healthy	4.98	2.11–11.74	0.0002
*Brevundimonas*	< 0.0001	Overweight vs healthy	8.72	3.24–23.48	< 0.0001
	< 0.0001	Unscheduled C-section vs vaginal	16.70	5.99–46.57	< 0.0001
		Scheduled C-section vs unscheduled C-section	0.071	0.011–0.46	0.0053
**3-month post-partum BMI**					
**Phylum**					
Actinobacteria	0.0058	Obese vs overweight	2.34	1.38–3.98	0.0017
		Obese vs healthy	2.02	1.18–3.46	0.010
**Genus**					
*Corynebacterium*	< 0.0001	Obese vs overweight	4.84	2.19–10.72	0.0001
		Obese vs healthy	7.77	2.95–20.43	< 0.0001
*Brevundimonas*	0.0005	Obese vs overweight	8.89	2.29–34.57	0.016
		Obese vs healthy	9.56	2.17–42.22	0.0029
	< 0.0001	Unscheduled C-section vs vaginal	13.01	4.01–42.20	< 0.0001
		Scheduled C-section vs unscheduled C-section	0.08	0.013–0.62	0.015

Separate Poisson regression models were run for pre-pregnancy BMI and 3-month post-partum BMI, while adjusting for maternal glucose tolerance status, mode of delivery, DNA extraction batch, and PCR sequencing batch. Statistically significant main group effect findings shown only (group effect: *p* ≤ 0.022 for phylum, *p* ≤ 0.017 for genus; pairwise comparison: *p* < 0.05). An interaction term between pre-pregnancy BMI and maternal glucose tolerance status was found to be statistically significant for *Gemella*. No other statistically significant interactions were found between maternal pre-pregnancy BMI and glucose tolerance status. *Gemella* showed an increased incidence among mothers with an overweight BMI (versus healthy BMI) and concurrent gestational diabetes IRR, CI (5.96 [1.85–19.21], *p* = 0.0028). An increased incidence of *Gemella* was also observed in mothers with an obese BMI and concurrent impaired glucose tolerance versus both mothers with overweight (11.42 [1.49–87.67], *p* = 0.019) and healthy weight BMIs (4.04 [1.63–10.01], *p* = 0.0026). *Abbreviations*: confidence interval *CI*, incidence rate ratio, *IRR*

### Association between maternal BMI and the milk microbiota

Pre-pregnancy BMI (i.e., healthy, overweight, obese) was found to be most consistently associated with differentially abundant taxa after controlling for relevant maternal characteristics. Mothers categorized as obese pre-pregnancy displayed a lower incidence of Proteobacteria (incidence rate ratio [IRR]: 0.62 [95% CI: 0.43–0.90]) in their milk as compared to mothers with an overweight BMI (Table 2). Conversely, mothers with overweight presented with an increased incidence of Proteobacteria in their milk, compared to healthy weight mothers (1.23 [1.00–1.50]). Mothers defined as obese pre-pregnancy had a greater incidence of Bacteroidetes in their milk as compared to mothers with an overweight (3.70 [1.61–8.48]) or healthy BMI (2.56 [1.27–5.17]). When examining 3-month post-partum BMI, Actinobacteria incidence was greater in women with obesity versus both mothers with an overweight (2.34 [1.38–3.98]) or healthy BMI (2.02 [1.18–3.46]).

At the genus-level, women with obesity pre-pregnancy displayed a higher incidence of *Staphylococcus* as compared to mothers with an overweight (2.50 [1.09–5.72]) or healthy BMI (3.15 [1.47–6.08]) (Table 2). Mothers with an obese BMI pre-pregnancy also displayed a greater incidence of *Corynebacterium* in their milk versus both mothers with an overweight (5.13 [1.79–14.70]) or healthy BMI (4.98 [2.11–11.74]). This same relationship with *Corynebacterium* was seen in mothers with obesity at 3-months post-partum compared to mothers with overweight (4.84 [2.19–10.72]) or a healthy BMI (7.77 [2.95–20.43]). An increased incidence of *Brevundimonas* was also observed in mothers with an overweight BMI pre-pregnancy versus healthy BMI (8.72 [3.24–23.48]). At 3-months post-partum, women with obesity also displayed a greater incidence of *Brevundimonas* versus both those with overweight (8.89 [2.29–34.57]) and those at a healthy weight (9.56 [2.17–42.22]) (Table 2).

### Association between maternal glucose tolerance status and the milk microbiota

When examining the interaction between BMI and maternal glucose tolerance status, *Gemella* showed an increased incidence among mothers with an overweight (versus healthy) BMI with gestational diabetes (5.96 [1.85–19.21]) (Additional file 11, Table S9). In addition, *Gemella* was increased in mother’s with obesity and concurrent impaired glucose tolerance versus both overweight (11.42 [1.49–87.67]) and healthy BMI (4.04 [1.63–10.01]) mothers with impaired glucose tolerance.

### Association between mode of delivery and the milk microbiota

Associations between mode of delivery and the differential abundance of select taxa at the phylum and genus level were found for both pre-pregnancy and post-partum BMI models (Table 2, Additional file 11, Table S9). A greater incidence of *Brevundimonas* was observed in mothers who underwent an unscheduled C-section versus a vaginal delivery from both the pre-pregnancy BMI model (16.70 [5.99–46.57]) and 3-month post-partum BMI model (13.01 [4.01–42.20]). Conversely, a reduced incidence of *Brevundimonas* was observed in mothers who underwent a scheduled C-section versus an unscheduled C-section in both the pre-pregnancy BMI model (0.071 [0.011–0.46]) and the 3-month post-partum BMI model (0.08 [0.013–0.62]).

### Association between maternal ethnicity and the milk microbiota

Lastly, ethnicity (white, Asian, other [South Asian, Black, other]) was associated with the differential abundance of *Corynebacterium*, *Brevundimonas* and *Aeromonas* (Table 3). White mothers had a reduced incidence of both *Corynebacterium* (0.27 [0.12–0.59]) and *Brevundimonas* (0.084 [0.015–0.46]) when compared to ‘other’ mothers and Asian mothers, respectively; Asian mothers also had a reduced incidence of *Corynebacterium* when compared to ‘other’ mothers (0.17 [0.049–0.63]).

**Table 3:** Associations between ethnicity and the top 5 phyla and top 10 genera

Taxa	Group effect *p*-value	Pairwise comparison	IRR	95% CI	Pairwise comparison *p*-value
**Genus**					
*Corynebacterium*	0.0008	White vs other	0.27	0.12–0.59	0.001
		Asian vs other	0.17	0.049–0.63	0.0075
*Brevundimonas*	0.0051	White vs Asian	0.084	0.015–0.46	0.0042
*Aeromonas*	0.022	Asian vs other	0.020	0.0007–0.59	0.023

Ethnicity was investigated for all taxa and models were adjusted for DNA extraction and PCR sequencing batch effects. Statistically significant findings shown only (group effect: *p* ≤ 0.022 for phylum, *p* ≤ 0.017 for genus; pairwise comparison: *p* < 0.05). No statistically significant associations were found between ethnicity and any phylum-level taxa. Other: pooled South Asian, Black, other. *Abbreviations*: confidence interval, *CI*, incidence rate ratio, *IRR*

### Association between maternal BMI, glucose tolerance status, mode of delivery, ethnicity and functional gene expression of the milk microbiota

We carried out functional inference analyses using Piphillin to assess if there were any differences in predicted functional capabilities of the milk microbiota based on the maternal clinical data. In contrast with the bacterial taxonomic results, the relative abundance of the 20 top KEGG pathways across all milk samples was fairly consistent (Additional file 12, Fig. S3). We analyzed the association between maternal clinical data and KEGG ortholog (KO) beta-diversity as well as examined the association between maternal clinical parameters and differentially-expressed KEGG pathways (Additional file 13, Table S10; Additional file 14, Table S11; Additional file 15, Table S12). No significant associations were found when examining metadata and KO beta-diversity (Additional file 13, Table S10); however, one statistically significant differentially-expressed set of pathways was observed (Additional file 14, Table S11; Additional file 16, Fig. S4). BMI, specifically the obese sub-category, was shown to be associated with enrichment of the KEGG pathway “Biosynthesis of secondary metabolites” (coefficient = 0.0024, *P*_FDR_ < 0.1) (Additional file 16, Fig. S4). Analysis of individual genes within this KEGG pathway and maternal metadata did not yield any statistically significant associations.

## Discussion

Our results suggest that maternal factors, and most consistently maternal pre-pregnancy BMI, are associated with the microbial composition of human milk. This is the first study to include maternal glucose tolerance status in the investigations of the association between maternal BMI and the milk microbiota (Additional file 1, Table S1). Gestational diabetes is associated with a number of negative health outcomes, including an increased risk of type 2 diabetes and metabolic syndrome in the mother. According to a recent systematic review and dose-response meta-analysis, the risk of GDM increases by 4% for every unit increase in BMI [25]. While some negative health outcomes associated with GDM are related to maternal adiposity and vice versa, others are thought to be independently related to chronically impaired glucose metabolism. Thus, it is important to investigate the impact of maternal BMI and GDM together on the composition of the milk microbiota. Our results show a significant interaction between pre-pregnancy BMI and gestational glucose intolerance on the differential abundance of *Gemella* in human milk. This suggests that associations between pre-pregnancy maternal BMI and the human milk microbiota are differentially impacted by maternal glucose tolerance status.

We did not find any differences in alpha-diversity based on our maternal characteristics; however, we did find statistically significant differences in beta-diversity, with human milk microbiota separating, or non-randomly clustering, based on pre-pregnancy BMI even after adjustment for other covariates (Additional file 5, Fig. S1; Additional file 6, Fig. S1; Additional file 7, Table S5). The human gut microbiota has been reported to cluster as a function of body size, but this has not yet been reported for the human milk microbiota [26]. Our results demonstrating an association between maternal BMI and microbial composition is consistent with other smaller scale studies on human milk microbiotas (Additional file 1, Table S1). Cabrera-Rubio et al. (2012) examined the association between maternal body size and the differential abundance of human milk genera in a study of healthy Finnish women (*n* = 18) [20]. They reported an increase in *Staphylococcus* in human milk collected from obese women, which mirrors the findings in our study (Additional file 8, Table S6).

Mode of delivery was also associated with changes in the human milk microbiota at both the phylum and genus levels (Table 2; Additional file 8, Table S6). For example, we observed greater differential abundance of *Staphylococcus* in human milk from women who underwent a scheduled C-section versus vaginal delivery (Additional file 8, Table S6). Our results are similar to that reported by Cabrera-Rubio et al. (2012, 2016). These two small cross-sectional cohorts of healthy Finnish women (n = 18, 10) showed a non-statistically significant increase in *Staphylococcus* in milk observed among women who delivered their infant via a scheduled C-section versus a vaginal delivery [19, 20]. The proposed mechanism whereby mode of delivery alters the milk microbiota is via the infant oral cavity, which is colonized during either vaginal delivery or C-section; from here, retrograde inoculation of bacteria can occur from the infant’s oral cavity into the mammary gland via the suckling process with direct breastfeeding [9, 27–29]. Understanding how an infant’s gut microbiota and overall health is impacted by differences in the milk microbiota based on mode of delivery remains uninvestigated. Future studies profiling the human milk microbiome, infant salivary microbiome and gut microbiome with repeated clinical follow-up over time would help answer these questions.

The results of our multi-ethnic cohort revealed associations between ethnicity and specific bacterial taxa. Ethnicity and/or geographic location have been shown to be factors in determining various microbiomes of the body including the gut, oral cavity, respiratory tract, skin, and urogenital tract [30]. Ethnicity and geographic location typically come with an overlay of dietary variation, making the impact of each variable challenging to separate. Only a few studies to date have assessed associations between ethnicity and the milk microbiota; however, the ethnic/geographic groups differed from our study as they generally examined Europe, Africa and the United States, making it challenging to compare findings (Table 3; Additional file 9, Table S7; Additional file 11, Table S9) [12, 21, 31, 32].

We used a functional inference approach to characterize the microbial genetic potential in human milk. In agreement with what has been reported for other human-associated microbiotas, the functional capacity of the human milk microbiota is more stable than its taxonomic composition [33]. We then assessed whether there were specific predicted pathways that were suggested to be associated with maternal characteristics and found that maternal pre-pregnancy BMI, specifically the obese sub-category, was significantly associated with an increase in the “Biosynthesis of secondary metabolites” predicted KEGG pathway. Microbes produce secondary metabolites, which are small, bioactive molecules, not necessary for growth or development but are instead involved in microbe-host or microbe-microbe interactions [34, 35]. Indeed, many of the genes in the biosynthesis of secondary metabolites pathway encode for the biosynthesis of antibiotics [36]. We assessed whether any individual genes (KO terms) involved in the predicted “Biosynthesis of secondary metabolites” pathway were significantly associated with BMI using MaAsLin2; however, no statistically significant associations were found. We hypothesize this may mean it is the culmination of many genes together that are leading to the enrichment of the “Biosynthesis of secondary metabolites” pathway, and not one individual gene. However, these observations require confirmation in future studies.

Human milk is considered a low biomass sample and, for this reason, may be more affected by sample processing than higher biomass samples, such as stool. To address this concern, we used PCoA plots to visualize clustering, or lack thereof, of our milk samples and negative controls (Additional file 17, Fig. S5). Our negative controls were seen to cluster away from the samples, suggesting that our results do not arise from technical contaminates, which was confirmed using Adonis analyses to statistically corroborate that our samples clustered away from the negative controls (Weighted UniFrac R^2^ = 0.07, *p* = 0.0001; Bray-Curtis R^2^ = 0.10, *p* = 0.0001, Additional file 17, Fig. S5).

Strengths of the current study include the varied ethnicity of women included, clinical examination via an OGTT, and enrichment of the cohort with women of varying body sizes who had abnormal glucose tolerance status. These strengths allowed for a more fulsome investigation using multivariable statistics to determine how each maternal factor is independently associated with the milk microbiome. Despite our larger sample size as compared to most earlier studies, we did not have sufficient sample size for adjustment of all covariates of interest (e.g., maternal diet) and thus were not able to perform the granular analyses needed to fully investigate the sub-groups within each metadata category. Future research should strive for larger sample sizes providing greater statistical power in order to capture more potential confounders. Additionally, although an acceptable sequencing depth was reached, rare but important microbes may still have been missed as our methods may not have been sensitive enough to pick up on certain important microbes. The compositional nature of data generated from next-generation sequencing also remains a challenge since it is limited to assessing relative (versus absolute) abundance; in other words, it holds the assumption that if one bacterial taxa increases, another bacterial taxa must conversely decrease, regardless of how the absolute abundance of these taxa may be changing [37–39]. Lastly, although interesting, the use of Piphillin to determine associations between metadata and predicted pathways is only hypothesis generating and requires confirmation using a combination of shotgun metagenomics, metabolomic profiling or comparison of matched human milk samples. Moreover, the microbes identified in the milk from the present study likely include bacteria from the mother’s skin microbiota as her breast was not disinfected prior to milk sampling. Practically, however, mothers do not disinfect their breast prior to pumping and storing milk for their infant, nor do they disinfect prior to breastfeeding. Therefore, the human milk microbiota as collected in the current study is likely a more accurate depiction of what the infant would receive. Finally, our study is limited by its cross-sectional analytic design and thus we cannot assess how the milk microbiota changes over time. It is possible that the associations we identified between maternal factors and microbial composition in milk are not transitory and change across the course of lactation.

## Conclusions

Our study found that human milk has a highly personalized microbiota with high inter-individual variability. Expressed human milk at both the phylum and genus levels appear to be related to maternal metabolic and obstetrical factors. Surprisingly, glucose tolerance status was significantly associated with fewer microbiota parameters than anticipated. Most consistently, maternal pre-pregnancy BMI, despite glucose tolerance status, was associated with the differential abundance of various taxa in human milk and potentially the production of bacterial secondary metabolites as well. To understand the clinical significance of these findings, future research should explore how differences in the microbial composition of human milk impact infant’s microbial colonization and overall health.

## Methods

### Study participants and design

To address the research objectives of this study, we used maternal metabolic and obstetrical health data along with bio-banked human milk samples available from a previously conducted prospective cohort study (ClinicalTrials.gov Identifier: NCT01405547); a detailed description of the study protocol has been previously published [40]. Pregnant women (*n* = 216) were recruited from outpatient clinics at Mount Sinai Hospital in Toronto, Canada and completed a 3-h 100 g OGTT between March 2009 and July 2010. In total, 117 women donated a milk sample at 3 months post-partum, with 113 samples available for this study (Additional file 18, Fig. S6). Women were eligible for inclusion in the original study if they were ≥ 20 years of age and had an intention to breastfeed. Exclusion criteria included pre-existing diabetes diagnosis, current use of insulin, or completion of an OGTT prior to recruitment [41]. By design, mothers were recruited from clinics which follow higher risk pregnancies with a greater risk of either GDM or IGT diagnosis.

### Collection of demographic, anthropometric and metabolic data

During the first study visit, which occurred in late pregnancy (30 weeks [95% CI: 25–33 weeks]), demographic and anthropometric data were collected (e.g. age, ethnicity, weight, height); mothers were asked to recall their pre-pregnancy weight. All pregnant women in Canada are screened for GDM by way of a 50 g glucose challenge test (GCT). If the plasma glucose concentration at 1-h post-glucose load is ≥7.8 mmol/L, the patient is then referred for a diagnostic OGTT. Contrary to standard obstetrical practice, all women completed a 3-h 100 g OGTT in the current study during their first study visit regardless of whether or not they completed a GCT. The OGTT involved having blood samples drawn at fasting, 30, 60, 90, 120- and 180-min post-glucose load. Women were then diagnosed with either GDM, IGT, or as normoglycemic based on the following glycemic thresholds: 1) GDM diagnosis = 2 or more of the following: fasting blood glucose ≥5.8 mmol/L, 1-h blood glucose ≥10.6 mmol/L, 2-h blood glucose ≥9.2 mmol/L, or 3-h blood glucose ≥8.1 mmol/L, or 2) IGT diagnosis would exceed only one of the previous thresholds, or 3) normoglycemic = normal OGTT [40].

### Human milk collection, processing and amplification

At the three-month post-partum research visit, mothers were asked to pump a complete breast expression of milk using a double electric breast pump (Medela Inc., Illinois, USA) with a sterile pumping kit. Mothers were instructed not to pump or breastfeed their infant for 2 h before the study visit. Samples of whole human milk were then divided into aliquots and stored at − 80 °C until the time of analyses.

DNA was extracted from human milk using the NucleoSpin Food DNA Isolation Kit (Macherey-Nagel, Pennsylvania, USA) according to manufacturer’s instructions with modifications as we have described previously [42]. Due to the small concentration of DNA in human milk, an elution buffer volume of 30 μL, instead of the recommended 100 μL, was used to ensure adequate DNA concentrations for downstream PCR.

PCR amplification of the V4 hypervariable region was performed using the forward primer (515F) 5’AATGATACGGCGACCACCGAGATCTACACTATGGTA ATTGTGTGCCAGCMGCCGCGGTAA and reverse primer (806R) 5’CAAGCAGA AGACGGCATACGAGATA GTCAGTCAGCCGGACTACHVGGGTWTCTAAT [43]. PCR reactions were set up following the manufacturer’s recommendations (Roche) including 12.5 μL of KAPA2G Robust HotStart ReadyMix, 1.5 μL of 10 μM forward and 1.5 μL of 10 μM reverse primer, 3.5 μL of sterile water and 6 μL of DNA. Amplification of the V4 hypervariable region of the 16S rRNA gene involved 28 cycles of PCR: 95 **°**C for 3 min, 25–30 cycles of 95 **°**C for 15 s, 50 **°**C for 15 s and 72 **°**C for 15 s, followed by a 5 min 72 **°**C extension (different numbers of cycles between PCR runs were adjusted for statistically). All amplifications were completed in triplicate and all amplicons were run on a 1% TBE agarose gel to ensure accurate amplification (amplicon size ~ 390 bp). A negative control without template DNA and a positive control with DNA from a known bacterial species (*Pseudomonas aeruginosa*) were also included to confirm the amplification quality. Bands of the same size and intensity were pooled and quantified to create the pooled sequence library. Purification of the pooled library was completed with AMPure XP beads (0.8X volume of beads to 1X volume of library DNA) following the manufacturer’s protocol. The purified library was quantified using the Qubit High Sensitivity DNA Kit (Thermo Fisher Scientific). The quantified library was loaded on an Illumina MiSeq and sequenced using the MiSeq-V2–300 cycle chemistry to generate 150 PE reads.

### Bioinformatics analyses

The raw paired end sequences from the MiSeq instrument have been deposited to the NCBI Sequence Read Archive (http://www.ncbi.nlm.nih.gov/sra) under accession number PRJNA516669. The UPARSE pipeline (USEARCH) was used for sequence analysis. Raw paired end sequences were assembled (−fastq_mergepairs; −fastq_merge_maxee =1.0), filtered (−fastq_filter; −fastq_maxee = 0.5) and sequences shorter than 225 base pairs were removed (−fastq_filter; −fastq_minlen 225) [44]. Sequences were then de-replicated and sorted using USEARCH (−derep_full; −sortybysize). Chimeric sequences in the OTUs were detected and removed using the Ribosomal Database Project (RDP) 16S gold database (USEARCH), while ensuring the number of false positive chimeras detected was minimized [45]. Sequences were then grouped together into Operational Taxonomic Units (OTUs) at 97% similarity (−usearch_global). Taxonomy was assigned to these OTUs (RDP 16S gold database) (−utax) and OTU fasta sequences were aligned using PyNast via a QIIME python script (align_seqs.py). A phylogenetic tree was assembled using the FastTree QIIME python script (make_phylogeny.py) [46].

### Data analysis and statistics

The phyloseq package (1.25.2) in R (version 3.4.1) was used to analyze microbiota composition [47]. OTUs that only appeared once or twice (singletons and doubletons) were removed and all OTUs were rarefied to 20,000 reads/sample prior to calculating relative abundances at different taxonomic levels, alpha-diversity and beta-diversity using phyloseq.

Statistically significant differences between the alpha diversities (Chao1/Shannon indices determined in R) and maternal metabolic or obstetrical characteristics were determined using multivariable linear regression models (PROC MIXED) in SAS version 9.4. Independent variables included in the models were: maternal BMI (healthy = 18.5–24.9 kg/m^2^, overweight = 25–29.9 kg/m^2^, obese= > 30 kg/m^2^), maternal glucose tolerance status (GDM, IGT, normoglycemic), mode of delivery (vaginal, unscheduled C-section, scheduled C-section), DNA extraction batch, and PCR sequencing batch. Separate statistical models were built using pre-pregnancy and 3-month post-partum BMI as covariates, due to concerns about collinearity. An interaction term between BMI and maternal glucose tolerance status was also tested in each model and removed if it was non-significant. Due to our sample size and the number of covariates we wished to test, separate models for ethnicity (white, Asian, other [South Asian, Black, other]) were run that adjusted for DNA extraction and PCR sequencing batches, but no other covariates. Multicollinearity was assessed between independent variables in all models, using a variance inflation cut-off of > 5. The significance level was set at *p* < 0.05. Of note, 6 mothers with a pre-pregnancy BMI between 18.0–18.4 kg/m^2^ were placed in the “healthy BMI” group for all analyses.

Beta diversities and principal coordinate analysis (PCoA) were also ascertained in phyloseq and statistical significance based on maternal characteristics was determined using the adonis function in vegan (version 2.5–3) [24]. Adonis assesses the amount of variation explained by each metadata variable, such as maternal BMI or glucose tolerance status; all variables were run individually and together in adonis to adjust for one another. The interaction term between BMI and glucose tolerance status was also tested and removed if non-significant. Four patient samples were missing the post-partum BMI data and thus we used their pre-pregnancy BMI for the post-partum analyses. Again, the significance level was set at *p* < 0.05.

Multivariable Poisson regression models (PROC GENMOD) were run in SAS version 9.4 to assess differential abundance at the phylum and genus levels based on maternal characteristics. The Benjamini-Yekutieli cut point approach was used to account for multiple testing. A *p* ≤ 0.022 at the phylum level (5 tests) and *p* ≤ 0.017 (10 tests) at the genus level were considered statistically significant for the overall group effect. If the overall group-adjusted *p*-value was significant, pairwise comparisons were conducted and a pairwise *p* < 0.05 was considered statistically significant.

### Piphillin: functional analysis of human milk microbiota

Piphillin, a metagenomics inference tool, was used to infer functional capabilities in milk samples (https://piphillin.secondgenome.com/) [48]. In this study, the Kyoto Encyclopedia of Genes and Genomes (KEGG; https://www.genome.jp/kegg/) was used as a reference database to retrieve gene copy numbers and create a gene feature table from the 16S rRNA sequence data. Statistically significant associations between maternal characteristics and KEGG pathways were assessed in three ways: 1) examining the association between maternal characteristics and KO beta-diversity using Adonis in R (*p* < 0.05), 2) investigating metadata associated with differentially-expressed predicted functional pathways using MaAsLin2 in R (*P*_FDR_ < 0.1) and 3) investigating metadata associated with individual genes (KO) found in the differentially expressed functional pathways from step 2.

## Supplementary information

**Additional file 1: Table S1.** 16S rRNA studies examining the association of maternal body mass index (BMI) and mode of delivery on the milk microbiota of women delivering healthy term-born infants. Note: Only studies in which mature milk was collected between 1 week - 6 months post-partum and BMI and mode of delivery were investigated were included to more accurately compare with our own findings.

**Additional file 2: Table S2.** The intersection of pre-pregnancy BMI and gestational glucose tolerance status.

**Additional file 3: Table S3.** Proportion of samples containing the top 5 phyla and top 10 genera.

**Additional file 4: Table S4.** The association between maternal characteristics and milk microbiota alpha-diversity. Pre-pregnancy BMI and post-partum BMI models were adjusted for maternal glucose tolerance status, mode of delivery, DNA extraction and PCR sequencing batches. Ethnicity was adjusted for DNA extraction and PCR sequencing batch effects.

**Additional file 5: Figure S1A-E.** Visual representation of the association between maternal characteristics and milk microbiota beta-diversity (weighted UniFrac metric). Principal coordinate analysis (PCoA, weighted UniFrac) plots comparing microbiota composition based on (A) maternal glucose tolerance, (B) mode of delivery, (C) pre-pregnancy BMI, (D) 3-month post-partum BMI, and (E) ethnicity. PCoA using the weighted UniFrac distance metric showed that microbiota profiles did not separate based on maternal clinical data. No statistically significant findings were observed between beta-diversity and glucose tolerance, mode of delivery, BMI, or ethnicity. Abbreviations: GDM, gestational diabetes mellitus, IGT, impaired glucose tolerance; CS, C-section.

**Additional file 6: Figure S2A-E.** Visual representation of the association between maternal characteristics and milk microbiota beta-diversity (Bray-Curtis metric). Principal coordinate analysis (PCoA, Bray-Curtis dissimilarity) plots comparing microbiota composition based on (A) maternal glucose tolerance, (B) mode of delivery, (C) pre-pregnancy BMI, (D) 3-month post-partum BMI, and (E) ethnicity. PCoA using the Bray-Curtis dissimilarity showed that microbiota profiles separated based on pre-pregnancy BMI (R^2^ = 0.037, *p* = 0.031), even after adjustment for maternal glucose tolerance status, mode of delivery, DNA extraction batch, and PCR sequencing batch. No statistically significant findings were observed between beta-diversity and glucose tolerance, mode of delivery, 3-month post-partum BMI or ethnicity. Abbreviations: GDM, gestational diabetes mellitus, IGT, impaired glucose tolerance; CS, C-section.

**Additional file 7: Table S5.** Examining the association between maternal characteristics and the milk microbiota beta-diversity. Statistically significant *p*-values (*p* < 0.05) are indicated with asterisks (*). ^1^Adjusted for maternal glucose tolerance status, mode of delivery, DNA extraction and PCR sequencing batches. Maternal glucose tolerance and mode of delivery were further adjusted for pre-pregnancy BMI. ^2^Ethnicity was adjusted for DNA extraction and PCR sequencing batch effects. An interaction term between BMI and glucose tolerance (Pre-pregnancy BMI-Maternal glucose tolerance status) was adjusted for batch effects and mode of delivery. The interaction term was found to be non-significant (as shown) and statistical models were re-run with it removed.

**Additional file 8: Table S6.** Associations between maternal characteristics and the top 5 phyla and top 10 genera: Results where pairwise comparisons were statistically significant but group effects were not. Separate Poisson regression models were run for pre-pregnancy BMI and 3-month post-partum BMI, while adjusting for maternal glucose tolerance status, mode of delivery, DNA extraction batch, and PCR sequencing batch. Statistically significant pairwise findings shown only (*p* < 0.05). Group effect thresholds [*p* ≤ 0.022 for phylum, *p* ≤ 0.017 for genus] were not significant, however, pairwise comparisons were (*p* < 0.05). All models were run testing an interaction term between BMI and maternal glucose tolerance status; this was removed from models if non-significant. Abbreviations: confidence interval, CI; incidence rate ratio, IRR; GDM, gestational diabetes.

**Additional file 9: Table S7.** Associations between ethnicity and the top 5 phyla and top 10 genera: Results where pairwise comparisons were statistically significant but group effects were not. Ethnicity was investigated for all taxa and models were adjusted for DNA extraction and PCR sequencing batch effects. Group effect thresholds [*p* ≤ 0.022 for phylum, *p* ≤ 0.017 for genus] were not significant, however, pairwise comparisons were (*p* < 0.05). No statistically significant associations were found between ethnicity and any phylum-level taxa. Abbreviations: confidence interval, CI; incidence rate ratio, IRR.

**Additional file 10: Table S8.** The association between maternal characteristics and the top 5 phyla. PreBMI, Pre-pregnancy BMI; PostBMI, Post-partum BMI.

**Additional file 11: Table S9.** The association between maternal characteristics and the top 10 genera. PreBMI, Pre-pregnancy BMI; PostBMI, Post-partum BMI.

**Additional file 12: Figure S3.** Relative abundance of top 20 KEGG pathways across milk samples.

**Additional file 13: Table S10.** Examining the association between maternal metadata and KEGG ortholog beta-diversity. Pre-pregnancy BMI and post-partum BMI models were adjusted for maternal glucose tolerance status, mode of delivery, DNA extraction and PCR sequencing batches. Maternal glucose tolerance and mode of delivery were further adjusted for pre-pregnancy BMI. Ethnicity was adjusted for DNA extraction and PCR sequencing batch effects. Statistical significance *p* < 0.05.

**Additional file 14: Table S11.** Statistically significant differentially-expressed predicted functional pathway. Pre-pregnancy BMI result was adjusted for maternal glucose tolerance status, mode of delivery, DNA extraction and PCR sequencing batches. BMI_cat = Pre-pregnancy BMI.

**Additional file 15: Table S12.** Complete results from MaAsLin2 (KEGG pathways). BMI_cat = Pre-pregnancy BMI.

**Additional file 16: Figure S4.** Statistically significant association between the KEGG pathway, “Biosynthesis of secondary metabolites”, and pre-pregnancy BMI (obese sub-category). BMI_cat = Pre-pregnancy BMI.

**Additional file 17: Figure S5.** Principal coordinate analysis (PCoA) plots examining negative controls compared to human milk samples. (A) Weighted UniFrac distances comparing microbiota composition based on negative controls and milk samples. (B) Bray Curtis dissimilarity comparing microbiota composition based negative controls and milk samples. Negative control = sterile water

**Additional file 18: Figure S6.** Flow diagram of subject participation at each time point.

## Data Availability

The sequences can be accessed at NCBI Sequence Read Archive (http://www.ncbi.nlm.nih.gov/sra) with accession number PRJNA516669. Patient clinical data are not publicly available to protect patient anonymity and confidentiality.

## Abbreviations

BMI: Body mass index
C-section: Caesarean delivery
CI: Confidence interval
GDM: Gestational diabetes mellitus
IGT: Impaired glucose tolerance
IRR: Incidence rate ratio
KEGG: Kyoto Encyclopedia of Genes and Genomes
KO: Kyoto Encyclopedia of Genes and Genomes (KEGG) ortholog
OGTT: Oral glucose tolerance test
OTU: Operational taxonomic unit
PCoA: Principal coordinates analysis
rRNA: Ribosomal RNA
SD: Standard deviation
