# Supplementary material for: Examining the relationship between maternal body size, gestational glucose tolerance status, mode of delivery and ethnicity on human milk microbiota at three months post-partum
Source: BMC Microbiol. 2020 Jul 20;20:219. doi: 10.1186/s12866-020-01901-9 (PMC7372813; doi:10.1186/s12866-020-01901-9)
Supplement: Supplementary file 1 — Additional file 1: Table S1. 16S rRNA studies examining the association of maternal body mass index (BMI) and mode of delivery on the milk microbiota of women delivering healthy term-born infants. Note: Only studies in which mature milk was collected between 1 week - 6 months post-partum and BMI and mode of delivery were investigated were included to more accurately compare with our own findings. [file 12866_2020_1901_MOESM1_ESM.docx]

**Table S1.** 16S rRNA studies examining the association of maternal body mass index (BMI) and mode of delivery on the milk microbiota of women delivering healthy term-born infants.

| Maternal factors | Sample size | Study location | Methods | Summary of results | Ref |
| --- | --- | --- | --- | --- | --- |
| Maternal BMI  Mode of delivery | 18 | Turku, Finland | -Iodine swab to disinfect breast  -Collected at 1-month post-partum, 6-months post-partum  -QIAamp DNA stool mini kit  -Hypervariable region: V1-V2  -454 pyrosequencing | -Mode of delivery associated with beta-diversity of human milk  -Normal body size associated with greater microbial diversity during the first month post-partum  -Increased *Staphylococcus*, reduced *Bifidobacterium* in mothers with higher BMI from milk collected at 6 months post-partum  -Reduced *Leuconostocaceae* and increased *Carnobacteriaceae* in scheduled C-section | (20) |
| Mode of delivery | 10 | Valencia, Spain | -Soap, water, chlorhexidine to disinfect breast  -First 500uL discarded  -Collected at 1-month post-partum  -QIAamp DNA stool mini kit  -Hypervariable region: V1-V3  -454 pyrosequencing | -Mode of delivery associated with beta-diversity of human milk  -C-section vs vaginal delivery associated with lower richness in human milk | (19) |
| Maternal BMI  Mode of delivery | 80 | China (Beijing), South Africa (Cape town), Finland (southwestern area), Spain (Valencia, Mediterranean area) | -Soap, water, chlorhexidine to disinfect breast  -Foremilk discarded  -Collected at 1-month post-partum  -InviMag stool DNA kit and bead beating with FastPrep  -Hypervariable region: V4  -Illumina MiSeq | -Firmicutes positively associated with BMI  -Vaginal delivery: Spanish women = highest Bacteroidetes; Chinese women = highest Actinobacteria  -Caesarean section: Spanish women = highest Proteobacteria and lower alpha-diversity | (21) |
| Mode of delivery | 39 | Ontario, Canada | -Sterile saline swab to disinfect breast  -Foremilk collected  -Collected at greater than 6 days post-partum  -QIAamp DNA stool kit  -Hypervariable region: V6  -Illumina MiSeq | -No statistically significant differences in beta-diversity or relative abundance between C-section (both elective and non-elective) and vaginal deliveries | (22) |
| Maternal BMI  Mode of delivery | 393 | Vancouver, Edmonton, Winnipeg and Toronto, Canada | -No disinfection of breast  -Mix of fore- and hindmilk from multiple feeds during 24-hour period  -Collected at 3-4 months post-partum  -Quick DNA fungal/bacterial extraction kit  -Hypervariable region: V4  -Illumina MiSeq | -Mode of delivery was not associated with overall bacterial richness and diversity in human milk  -Maternal BMI inversely associated with Proteobacteria diversity and positively associated with Firmicutes diversity | (23) |

Note: Only studies in which mature milk was collected between 1 week – 6 months post-partum and BMI and mode of delivery were investigated were included to more accurately compare with our own findings.
