# Supplementary material for: Examining the relationship between maternal body size, gestational glucose tolerance status, mode of delivery and ethnicity on human milk microbiota at three months post-partum
Source: BMC Microbiol. 2020 Jul 20;20:219. doi: 10.1186/s12866-020-01901-9 (PMC7372813; doi:10.1186/s12866-020-01901-9)
Supplement: Supplementary file 3 — Additional file 3: Table S3. Proportion of samples containing the top 5 phyla and top 10 genera. [file 12866_2020_1901_MOESM3_ESM.docx]

**Table S3.** Proportion of samples containing the top 5 phyla and top 10 genera.

| Taxa | Proportion of samples containing taxa, No. %, (n= 109) |
| --- | --- |
| Phylum  Proteobacteria | 109 (100%) |
| Firmicutes | 109 (100%) |
| Actinobacteria | 109 (100%) |
| Bacteroidetes | 109 (100%) |
| Fusobacteria | 84 (77%) |
| *Genus*  *Pseudomonas* | 109 (100%) |
| *Streptococcus* | 109 (100%) |
| *Staphylococcus* | 109 (100%) |
| *Acinetobacter* | 109 (100%) |
| *Veillonella* | 109 (100%) |
| *Corynebacterium* | 108 (99%) |
| *Rothia* | 107 (98%) |
| *Gemella* | 106 (97%) |
| *Brevundimonas* | 105 (96%) |
| *Aeromonas* | 48 (44%) |
