# Supplementary figures and images for: Examining the relationship between maternal body size, gestational glucose tolerance status, mode of delivery and ethnicity on human milk microbiota at three months post-partum
Source: BMC Microbiol. 2020 Jul 20;20:219. doi: 10.1186/s12866-020-01901-9 (PMC7372813; doi:10.1186/s12866-020-01901-9)

## Slide 1
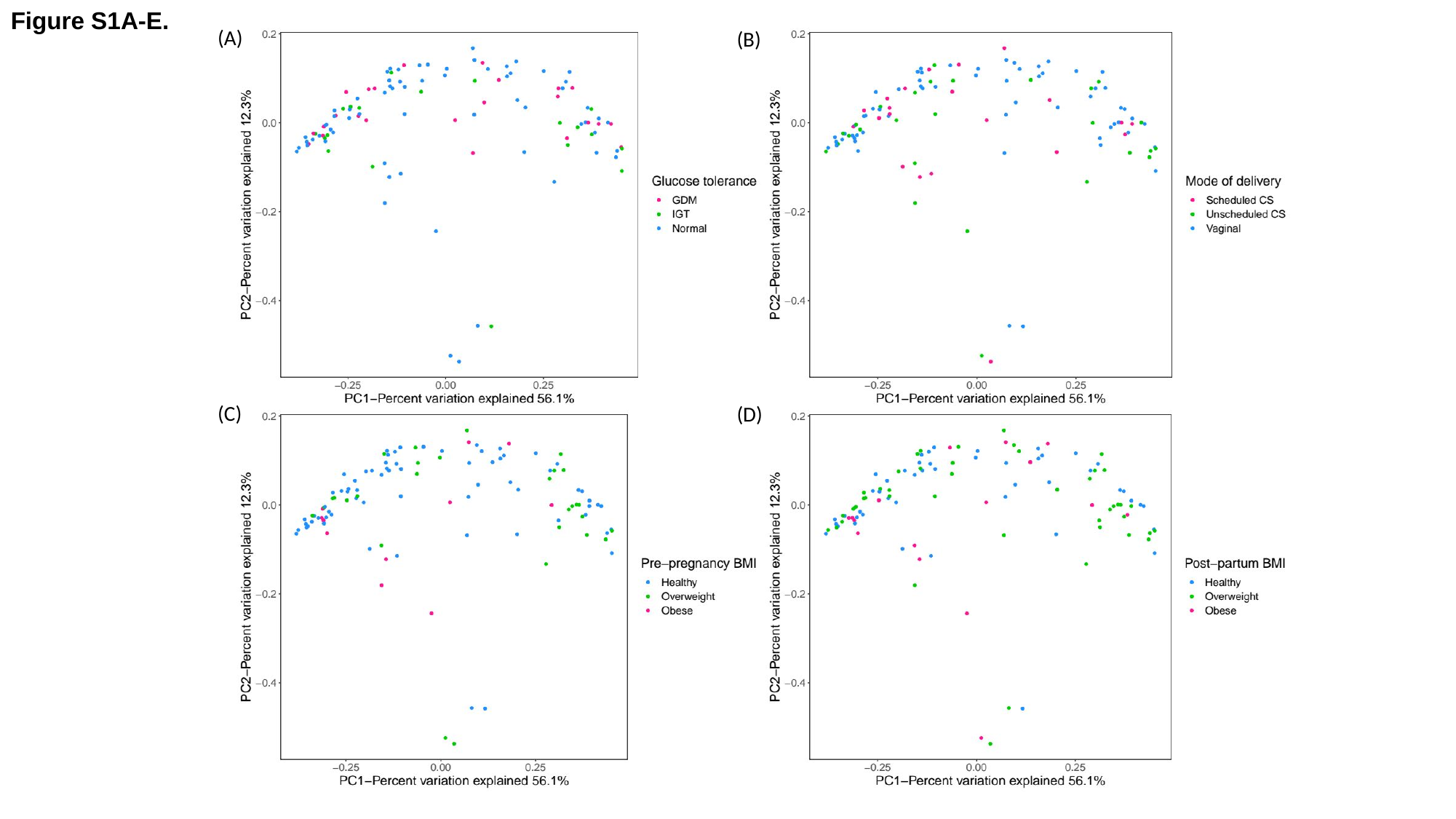

Figure S1A-E.
(A)
(B)
(C)
(D)

## Slide 2
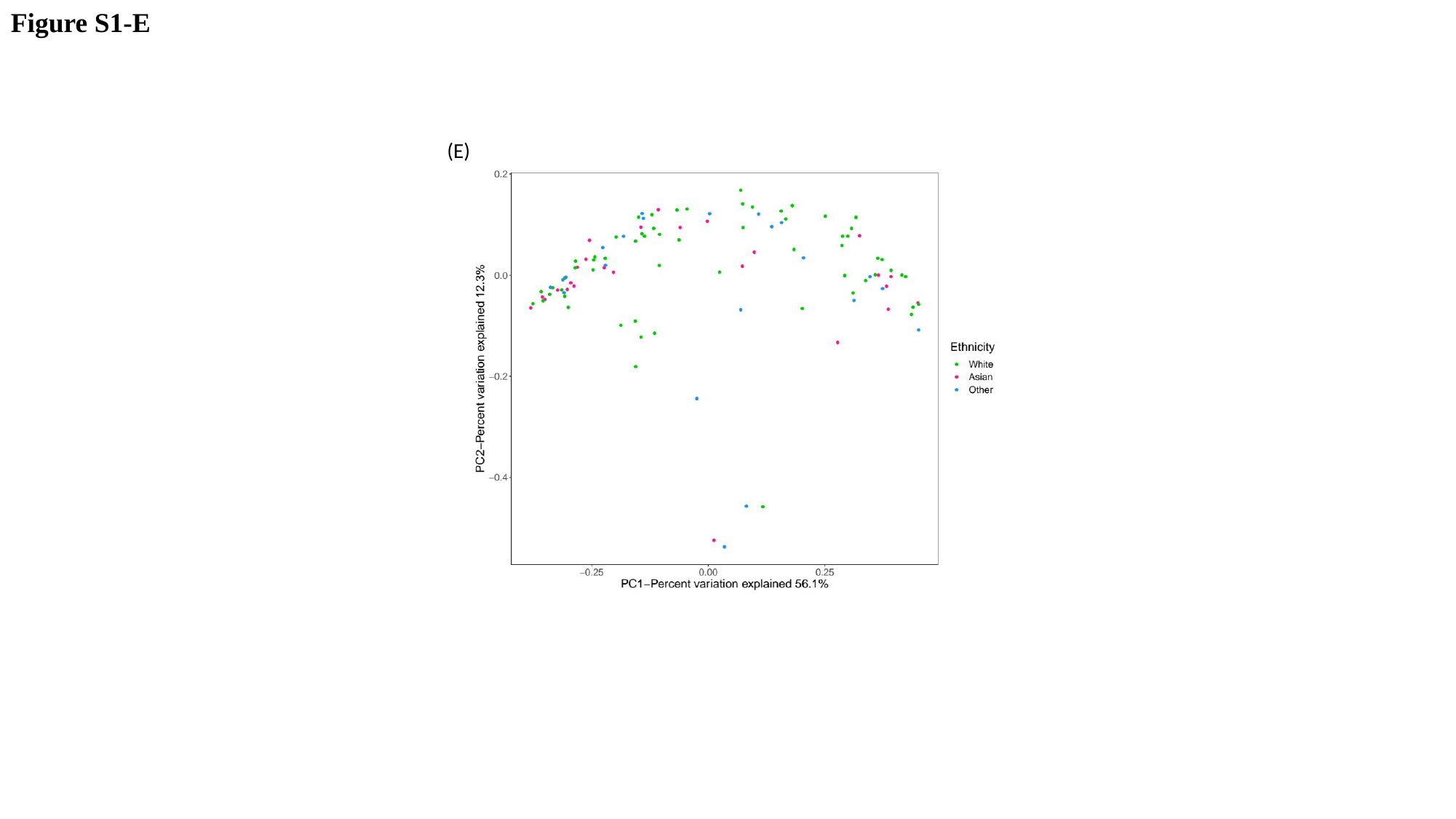

Figure S1-E
(E)

Supplement: Supplementary file 5 — Additional file 5: Figure S1A-E. Visual representation of the association between maternal characteristics and milk microbiota beta-diversity (weighted UniFrac metric). Principal coordinate analysis (PCoA, weighted UniFrac) plots comparing microbiota composition based on (A) maternal glucose tolerance, (B) mode of delivery, (C) pre-pregnancy BMI, (D) 3-month post-partum BMI, and (E) ethnicity. PCoA using the weighted UniFrac distance metric showed that microbiota profiles did not separate based on maternal clinical data. No statistically significant findings were observed between beta-diversity and glucose tolerance, mode of delivery, BMI, or ethnicity. Abbreviations: GDM, gestational diabetes mellitus, IGT, impaired glucose tolerance; CS, C-section. [file 12866_2020_1901_MOESM5_ESM.pptx]

## Slide 1
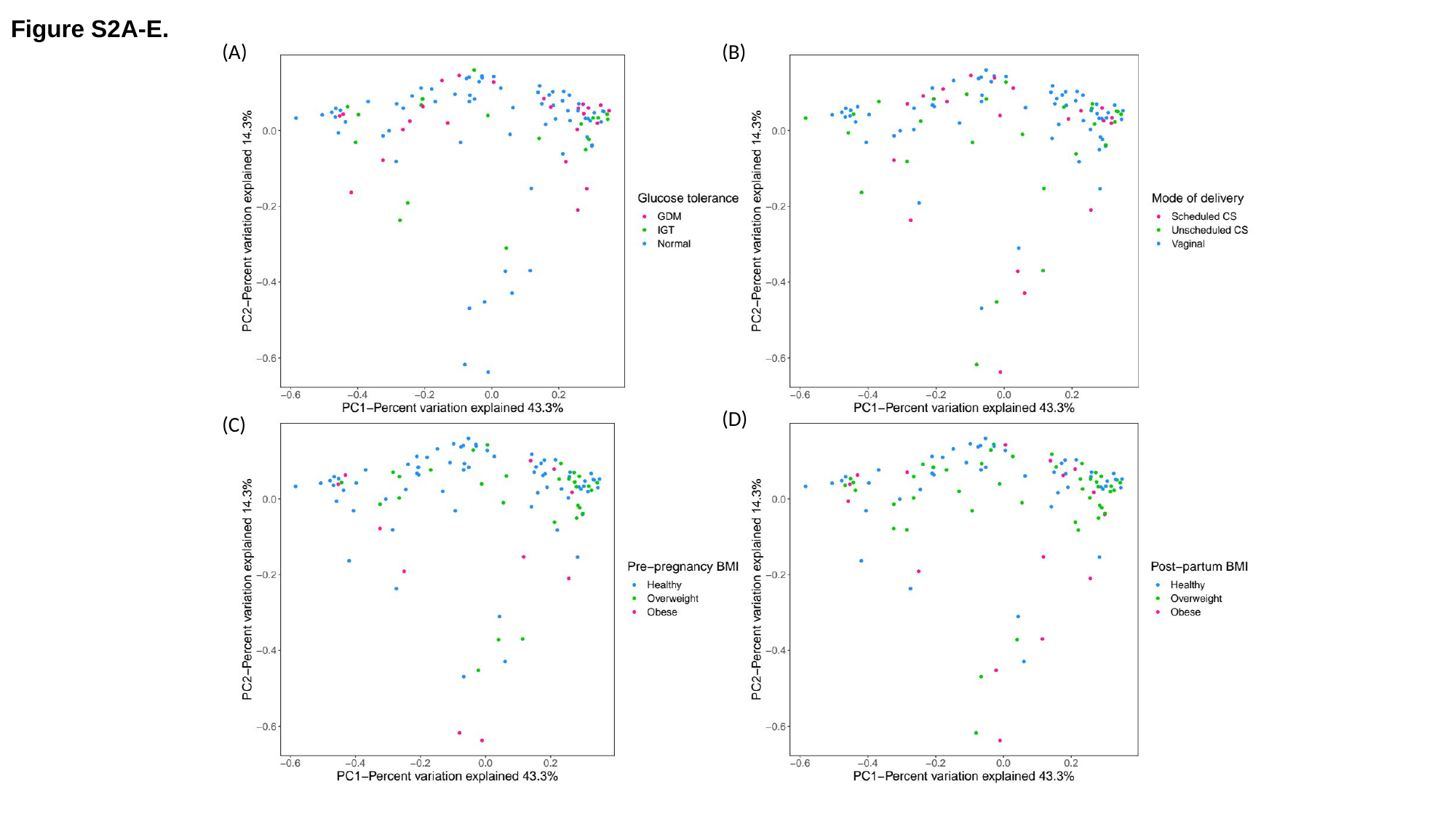

Figure S2A-E.
(A)
(B)
(D)
(C)

## Slide 2
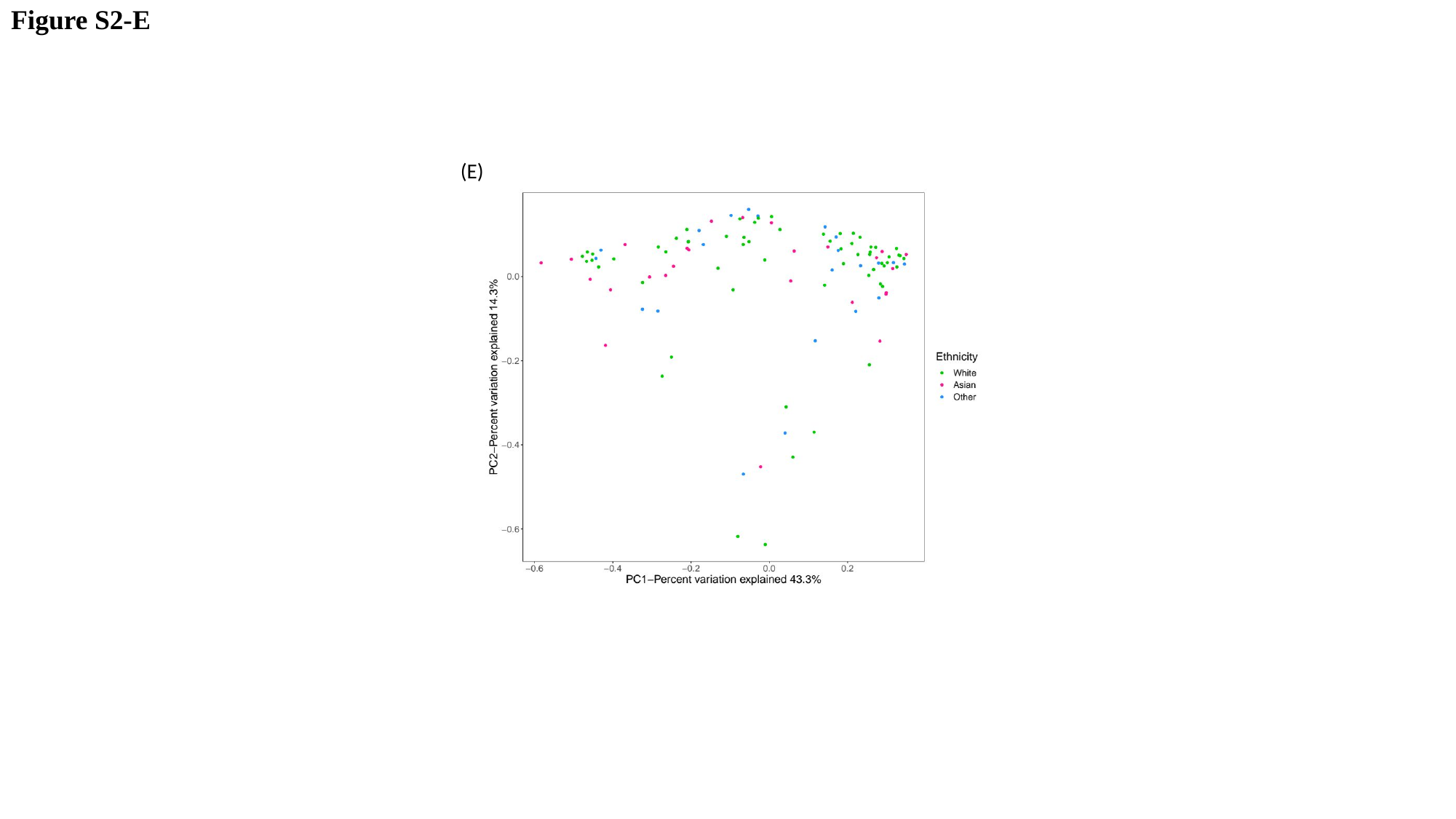

# Figure S2-E
(E)

Supplement: Supplementary file 6 — Additional file 6: Figure S2A-E. Visual representation of the association between maternal characteristics and milk microbiota beta-diversity (Bray-Curtis metric). Principal coordinate analysis (PCoA, Bray-Curtis dissimilarity) plots comparing microbiota composition based on (A) maternal glucose tolerance, (B) mode of delivery, (C) pre-pregnancy BMI, (D) 3-month post-partum BMI, and (E) ethnicity. PCoA using the Bray-Curtis dissimilarity showed that microbiota profiles separated based on pre-pregnancy BMI (R2 = 0.037, p = 0.031), even after adjustment for maternal glucose tolerance status, mode of delivery, DNA extraction batch, and PCR sequencing batch. No statistically significant findings were observed between beta-diversity and glucose tolerance, mode of delivery, 3-month post-partum BMI or ethnicity. Abbreviations: GDM, gestational diabetes mellitus, IGT, impaired glucose tolerance; CS, C-section. [file 12866_2020_1901_MOESM6_ESM.pptx]

## Slide 1
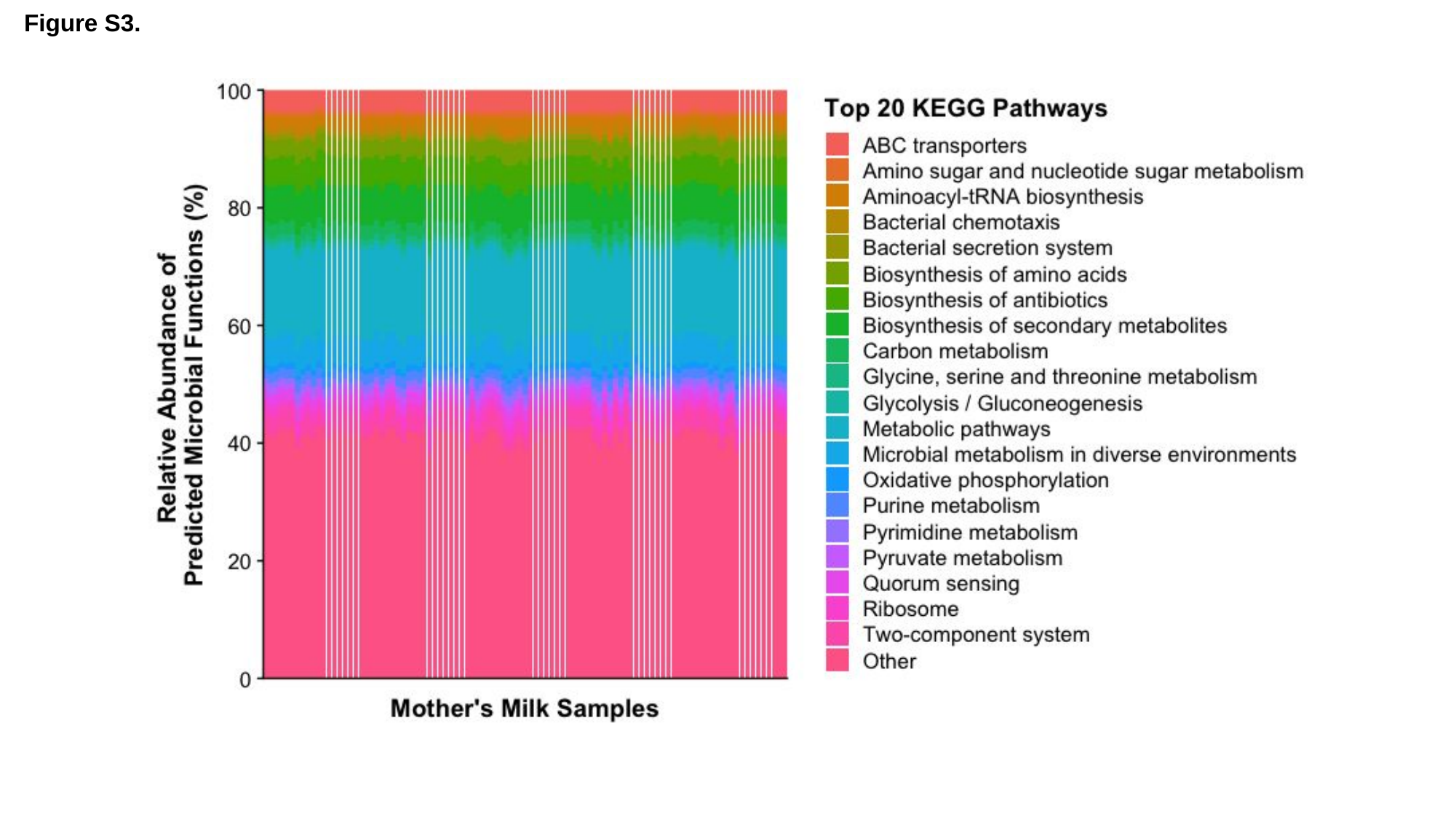

# Figure S3.

Supplement: Supplementary file 12 — Additional file 12: Figure S3. Relative abundance of top 20 KEGG pathways across milk samples. [file 12866_2020_1901_MOESM12_ESM.pptx]

## Slide 1
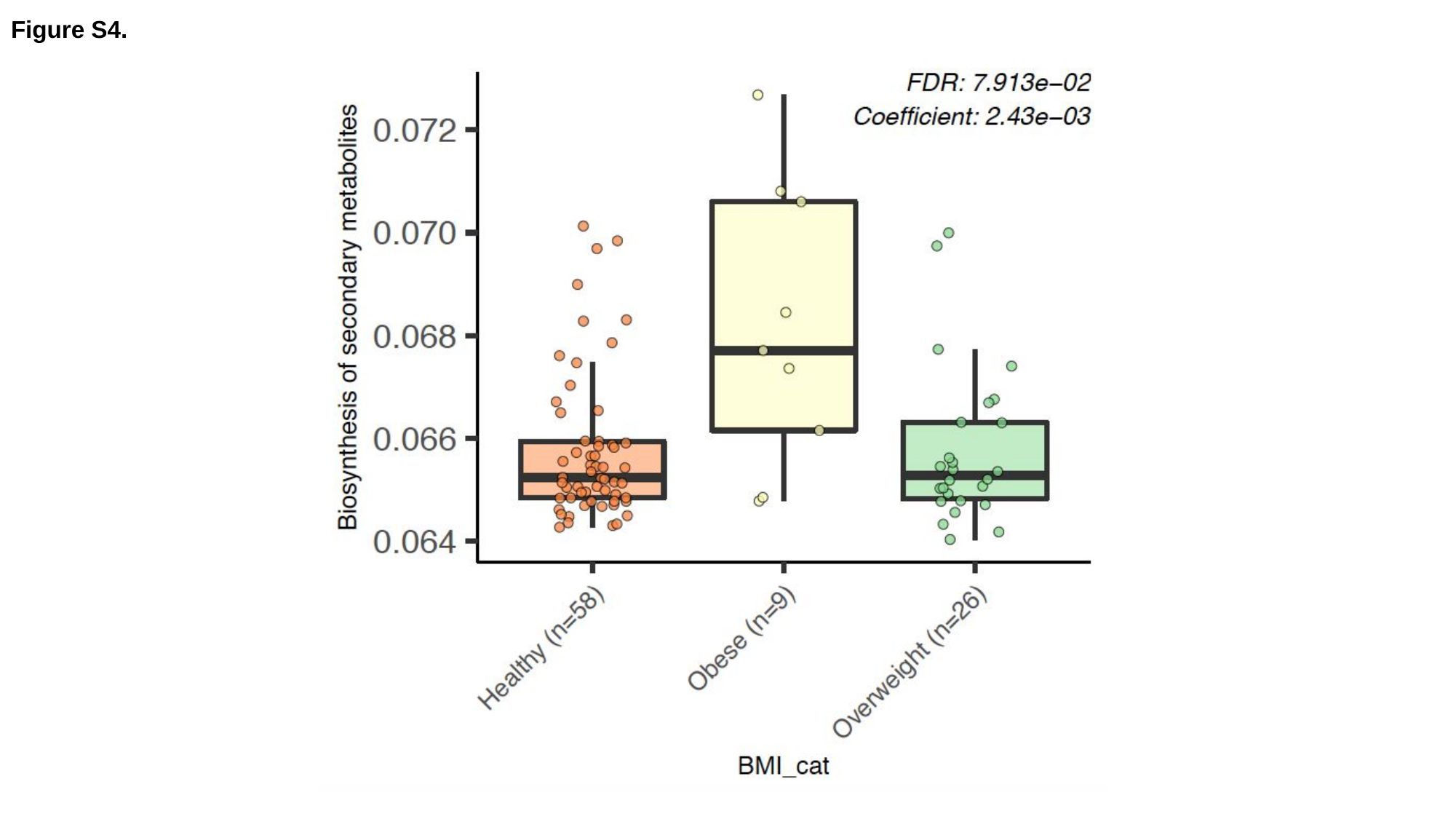

# Figure S4.

Supplement: Supplementary file 16 — Additional file 16: Figure S4. Statistically significant association between the KEGG pathway, “Biosynthesis of secondary metabolites”, and pre-pregnancy BMI (obese sub-category). BMI_cat = Pre-pregnancy BMI. [file 12866_2020_1901_MOESM16_ESM.pptx]

## Slide 1
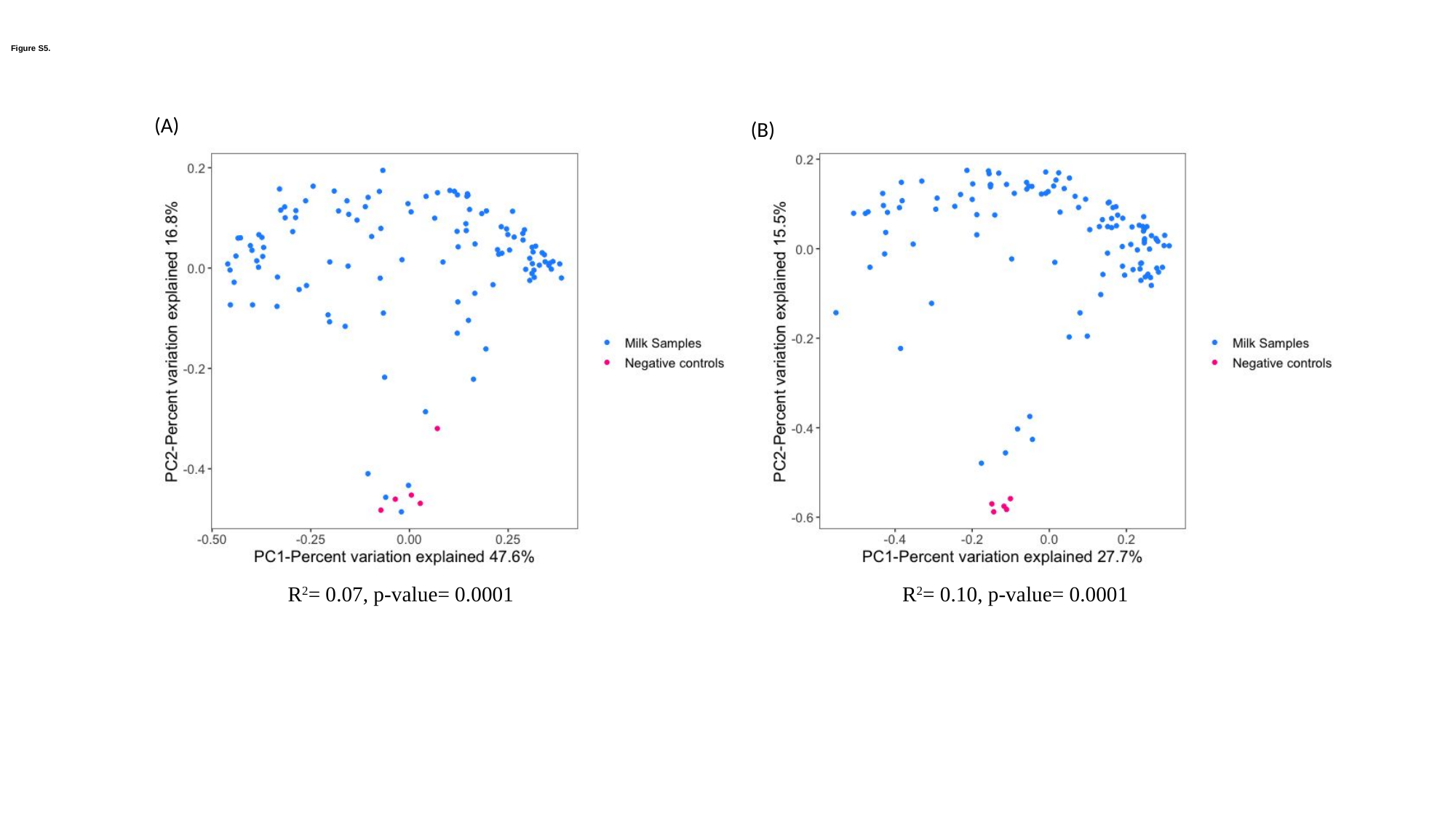

# Figure S5.
(A)
(B)
R2= 0.10, p-value= 0.0001
R2= 0.07, p-value= 0.0001

Supplement: Supplementary file 17 — Additional file 17: Figure S5. Principal coordinate analysis (PCoA) plots examining negative controls compared to human milk samples. (A) Weighted UniFrac distances comparing microbiota composition based on negative controls and milk samples. (B) Bray Curtis dissimilarity comparing microbiota composition based negative controls and milk samples. Negative control = sterile water [file 12866_2020_1901_MOESM17_ESM.pptx]
