# Supplementary material for: Examining the relationship between maternal body size, gestational glucose tolerance status, mode of delivery and ethnicity on human milk microbiota at three months post-partum
Source: BMC Microbiol. 2020 Jul 20;20:219. doi: 10.1186/s12866-020-01901-9 (PMC7372813; doi:10.1186/s12866-020-01901-9)
Supplement: Supplementary file 7 — Additional file 7: Table S5. Examining the association between maternal characteristics and the milk microbiota beta-diversity. Statistically significant p-values (p < 0.05) are indicated with asterisks (*). 1Adjusted for maternal glucose tolerance status, mode of delivery, DNA extraction and PCR sequencing batches. Maternal glucose tolerance and mode of delivery were further adjusted for pre-pregnancy BMI. 2Ethnicity was adjusted for DNA extraction and PCR sequencing batch effects. An interaction term between BMI and glucose tolerance (Pre-pregnancy BMI-Maternal glucose tolerance status) was adjusted for batch effects and mode of delivery. The interaction term was found to be non-significant (as shown) and statistical models were re-run with it removed. [file 12866_2020_1901_MOESM7_ESM.docx]

| Characteristic | Weighted UniFrac | | Bray-Curtis | |
| --- | --- | --- | --- | --- |
|  | R^2^ | *p*-value | R^2^ | *p*-value |
| **Unadjusted** |  |  |  |  |
| Pre-pregnancy BMI | 0.040 | 0.045* | 0.045 | 0.014* |
| 3-month post-partum BMI | 0.016 | 0.50 | 0.022 | 0.28 |
| Maternal glucose tolerance | 0.0076 | 0.94 | 0.0072 | 0.99 |
| Mode of delivery | 0.014 | 0.58 | 0.014 | 0.65 |
| Ethnicity | 0.012 | 0.70 | 0.016 | 0.51 |
| Pre-pregnancy BMI−Maternal glucose tolerance status | 0.040 | 0.32 | 0.049 | 0.13 |
| **Adjusted**^1^ |  |  |  |  |
| Pre-pregnancy BMI | 0.035 | 0.066 | 0.037 | 0.031* |
| 3-month post-partum BMI | 0.017 | 0.48 | 0.020 | 0.32 |
| Maternal glucose tolerance | 0.0079 | 0.91 | 0.0076 | 0.96 |
| Mode of delivery | 0.013 | 0.66 | 0.0096 | 0.89 |
| ^2^Ethnicity | 0.014 | 0.57 | 0.016 | 0.46 |
| Pre-pregnancy BMI−Maternal glucose tolerance status | 0.038 | 0.38 | 0.047 | 0.14 |
| Residuals (Pre-pregnancy BMI) |  |  | 0.86 |  |

**Table S5.** Examining the association between maternal characteristics and the milk microbiota

beta-diversity.

Statistically significant p-values (*p*<0.05) are indicated with asterisks (*). ^1^Adjusted for maternal glucose tolerance status, mode of delivery, DNA extraction and PCR sequencing batches. Maternal glucose tolerance and mode of delivery were further adjusted for pre-pregnancy BMI. ^2^Ethnicity was adjusted for DNA extraction and PCR sequencing batch effects. An interaction term between BMI and glucose tolerance (Pre-pregnancy BMI−Maternal glucose tolerance status) was adjusted for batch effects and mode of delivery. The interaction term was found to be non-significant (as shown) and statistical models were re-run with it removed.
