# Supplementary material for: Examining the relationship between maternal body size, gestational glucose tolerance status, mode of delivery and ethnicity on human milk microbiota at three months post-partum
Source: BMC Microbiol. 2020 Jul 20;20:219. doi: 10.1186/s12866-020-01901-9 (PMC7372813; doi:10.1186/s12866-020-01901-9)
Supplement: Supplementary file 8 — Additional file 8: Table S6. Associations between maternal characteristics and the top 5 phyla and top 10 genera: Results where pairwise comparisons were statistically significant but group effects were not. Separate Poisson regression models were run for pre-pregnancy BMI and 3-month post-partum BMI, while adjusting for maternal glucose tolerance status, mode of delivery, DNA extraction batch, and PCR sequencing batch. Statistically significant pairwise findings shown only (p < 0.05). Group effect thresholds [p ≤ 0.022 for phylum, p ≤ 0.017 for genus] were not significant, however, pairwise comparisons were (p < 0.05). All models were run testing an interaction term between BMI and maternal glucose tolerance status; this was removed from models if non-significant. Abbreviations: confidence interval, CI; incidence rate ratio, IRR; GDM, gestational diabetes. [file 12866_2020_1901_MOESM8_ESM.docx]

**Table S6.** Associations between maternal characteristics and the top 5 phyla and top 10 genera: Results where pairwise comparisons were statistically significant but group effects were not.

| Taxa | Group effect  *p*-value | Pairwise comparison | IRR | 95% CI | Pairwise comparison  *p*-value |
| --- | --- | --- | --- | --- | --- |
| Pre-pregnancy BMI | | | | | |
| Phylum |  |  |  |  |  |
| Firmicutes | 0.060 | Obese vs overweight | 1.76 | 1.06-2.94 | 0.030 |
| Actinobacteria | 0.039 | Obese vs overweight | 2.23 | 1.19-4.17 | 0.012 |
|  |  | Obese vs healthy | 1.76 | 1.01-3.04 | 0.045 |
| Bacteroidetes | 0.070 | GDM vs normoglycemia | 0.34 | 0.14-0.85 | 0.021 |
|  | 0.066 | Scheduled C-section vs vaginal | 2.16 | 1.12-4.14 | 0.021 |
| Genus |  |  |  |  |  |
| *Pseudomonas* | 0.055 | Obese vs overweight | 0.63 | 0.41-0.96 | 0.032 |
| *Streptococcus* | 0.083 | Overweight vs healthy | 0.59 | 0.37-0.95 | 0.029 |
| *Staphylococcus* | 0.060 | Scheduled C-section vs vaginal | 2.43 | 1.14-5.21 | 0.022 |
| *Veillonella* | 0.029 | Obese vs overweight | 3.88 | 1.25-12.11 | 0.019 |
|  |  | Obese vs healthy | 2.91 | 1.18-7.14 | 0.020 |
| 3-month post-partum BMI | | | | | |
| Phylum |  |  |  |  |  |
| Bacteroidetes | 0.10 | Scheduled C-section vs vaginal | 2.16 | 1.05-4.44 | 0.037 |
| Genus |  |  |  |  |  |
| *Staphylococcus* | 0.023 | Obese vs overweight  Obese vs healthy | 2.59  2.40 | 1.25-5.38  1.12-5.14 | 0.011  0.024 |
| *Veillonella*  *Brevundimonas* | 0.029  0.071  0.038 | Scheduled C-section vs vaginal  Obese vs overweight  GDM vs normoglycemic | 2.62  5.33  0.12 | 1.29-5.35  1.25-22.62  0.022-0.68 | 0.0079  0.023  0.016 |

Separate Poisson regression models were run for pre-pregnancy BMI and 3-month post-partum BMI, while adjusting for maternal glucose tolerance status, mode of delivery, DNA extraction batch, and PCR sequencing batch. Statistically significant pairwise findings shown only (*p*<0.05). Group effect thresholds [*p*≤0.022 for phylum, *p*≤0.017 for genus] were not significant, however, pairwise comparisons were (*p*<0.05). All models were run testing for an interaction term between BMI and maternal glucose tolerance status- this was removed if non-significant. Abbreviations: confidence interval, CI; incidence rate ratio, IRR; GDM, gestational diabetes.
