# Supplementary material for: Examining the relationship between maternal body size, gestational glucose tolerance status, mode of delivery and ethnicity on human milk microbiota at three months post-partum
Source: BMC Microbiol. 2020 Jul 20;20:219. doi: 10.1186/s12866-020-01901-9 (PMC7372813; doi:10.1186/s12866-020-01901-9)
Supplement: Supplementary file 9 — Additional file 9: Table S7. Associations between ethnicity and the top 5 phyla and top 10 genera: Results where pairwise comparisons were statistically significant but group effects were not. Ethnicity was investigated for all taxa and models were adjusted for DNA extraction and PCR sequencing batch effects. Group effect thresholds [p ≤ 0.022 for phylum, p ≤ 0.017 for genus] were not significant, however, pairwise comparisons were (p < 0.05). No statistically significant associations were found between ethnicity and any phylum-level taxa. Abbreviations: confidence interval, CI; incidence rate ratio, IRR. [file 12866_2020_1901_MOESM9_ESM.docx]

**Table S7.** Associations between ethnicity and the top 5 phyla and top 10 genera: Results where pairwise comparisons were statistically significant but group effects were not.

| Taxa | Group effect p-value | Pairwise comparison | IRR | 95% CI | Pairwise comparison p-value |
| --- | --- | --- | --- | --- | --- |
| Genus |  |  |  |  |  |
| *Gemella* | 0.073 | White vs Asian | 0.45 | 0.22-0.95 | 0.037 |

Ethnicity was investigated for all taxa and models were adjusted for DNA extraction and PCR sequencing batch effects. Group effect thresholds [*p*≤0.022 for phylum, *p*≤0.017 for genus] were not significant, however, pairwise comparisons were (*p*<0.05). No statistically significant associations were found between ethnicity and any phylum-level taxa. Abbreviations: confidence interval, CI; incidence rate ratio, IRR.
