# Supplementary material for: Examining the relationship between maternal body size, gestational glucose tolerance status, mode of delivery and ethnicity on human milk microbiota at three months post-partum
Source: BMC Microbiol. 2020 Jul 20;20:219. doi: 10.1186/s12866-020-01901-9 (PMC7372813; doi:10.1186/s12866-020-01901-9)
Supplement: Supplementary file 18 — Additional file 18: Figure S6. Flow diagram of subject participation at each time point. [file 12866_2020_1901_MOESM18_ESM.pptx]

## Slide 1
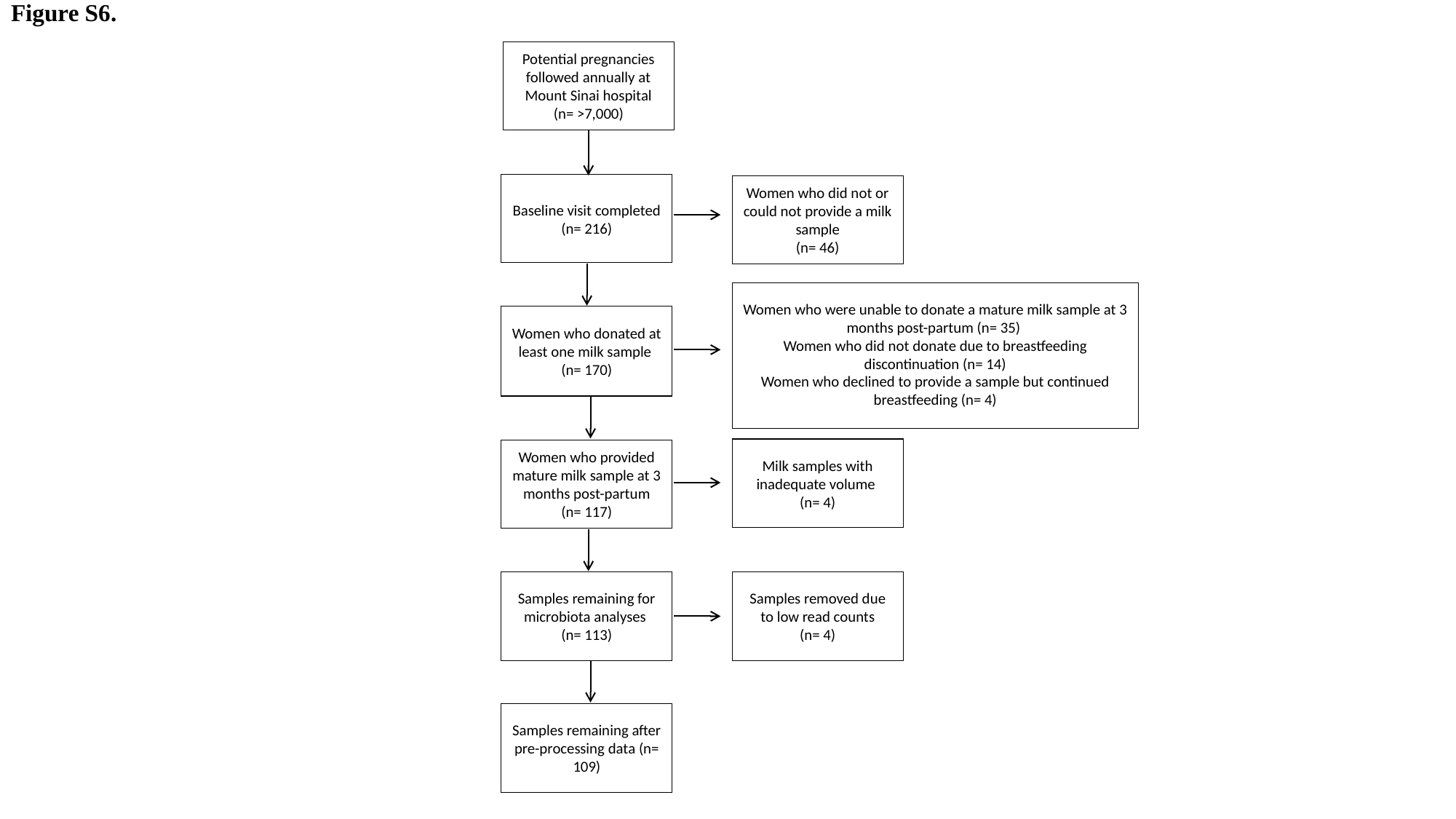

# Figure S6.
Potential pregnancies followed annually at Mount Sinai hospital
(n= >7,000)
Baseline visit completed
(n= 216)
Women who provided mature milk sample at 3 months post-partum
(n= 117)
Samples remaining for microbiota analyses
(n= 113)
Samples remaining after pre-processing data (n= 109)
Women who did not or could not provide a milk sample
(n= 46)
Women who were unable to donate a mature milk sample at 3 months post-partum (n= 35)
Women who did not donate due to breastfeeding discontinuation (n= 14)
Women who declined to provide a sample but continued breastfeeding (n= 4)
Milk samples with inadequate volume
(n= 4)
Samples removed due to low read counts
(n= 4)
Women who donated at least one milk sample
(n= 170)
